# Kaposi Sarcoma: A Rare Presentation of Elephantiasis Nostras Verrucosa

**DOI:** 10.7759/cureus.37339

**Published:** 2023-04-09

**Authors:** Andrew V Doodnauth, Jordan Zhou, Sigogini Sivarajah, Hannah E Xavier, Samy I. McFarlane

**Affiliations:** 1 Internal Medicine, Downstate Health Sciences University, Brooklyn, USA; 2 Internal Medicine, Downstate Health Sciences University, St. George's University, Brooklyn, USA

**Keywords:** chronic lymphedema, acquired immunodeficiency syndrome, human immunodeficiency virus, elephantiasis nostras verrucosa, kaposi sarcoma

## Abstract

Although a low-grade vascular tumor, Kaposi sarcoma (KS) can have mucosal, and visceral involvement. Additionally, disfiguring disseminated lesions can be seen in patients with human immunodeficiency virus (HIV) and acquired immunodeficiency syndrome (AIDS). KS may cause lymphatic obstruction leading to chronic lymphedema that further contributes to progressive cutaneous hypertrophy and severe disfigurement in the form of non-filarial elephantiasis nostras verrucosa (ENV).

This report highlights a case of a 33-year-old male with AIDS who presented in acute respiratory distress with bilateral lower extremity nodular lesions. We confirmed a diagnosis of KS with overlying ENV via a multi-disciplinary approach. Collaboratively, we optimized our patient and observed adequate treatment response and overall improvement in clinical status.

Our report emphasizes the importance of a multi-disciplinary approach in recognizing a rare presentation of ENV. Recognition of the disease and understanding the extent of the disease are crucial in preventing irreversible disease progression and allowing for maximum response.

## Introduction

Kaposi sarcoma (KS) is a vascular tumor that develops through the infection of human herpesvirus 8 (HHV-8) [1]. Risk factors for KS include non-adherence to highly active antiretroviral therapy (HAART) and identifying as an HIV-positive male homosexual [2]. It is a low-grade tumor and its aggressiveness is dependent on its epidemiology which is typically categorized as classic, African, acquired immunodeficiency syndrome (AIDS)-associated, and iatrogenic along with host immunity. AIDS-associated KS is particularly aggressive and usually presents with disseminated lesions which include mucosal and visceral involvement [1]. Patients with HIV and KS may also present with a relatively new aggressive syndrome with a high mortality rate - KS inflammatory cytokine syndrome [3].

Elephantiasis nostras verrucosa (ENV), a very rare presentation of severe disfigurement and lymphedema, may occur in AIDS-associated KS. Elephantiasis is typically known to be transmitted by mosquitoes carrying filarial nematodes, specifically, Wuchereria bancrofti, however, it can manifest in a patient with AIDS caused by lymphatic obstruction, leading to progressive cutaneous hypertrophy [4].

We report a case of a 33-year-old male with known human immunodeficiency virus (HIV), who presented with acute respiratory distress with severe bilateral lower extremity disfigurement. Further workup revealed a rare case of non-filarial ENV in the setting of KS.

## Case presentation

A 33-year-old male presented to the emergency department (ED) with worsening shortness of breath, pelvic pain, and foul-smelling, brown-colored fluid leakage from both lower extremities. Past medical history (PMHx) was significant for HIV on highly active antiretroviral therapy (HAART), asthma, bipolar disorder, and KS. Chart review was significant for a CD4+ count of 148 with a viral load of 185,000 seven months prior to this hospital course. On admission, the patient’s CD4+ count was 97 with an undetectable viral load. Upon further history, the patient described the lower extremity lesions to be chronic, but progressively getting worse over the last few months and stated that the shortness of breath had started only one day ago, which prompted his visit. He also endorsed fever, chills, night sweats, and pleuritic chest pain. There were no other pertinent findings after a thorough review of all organ systems.

On arrival, vitals were as follows: blood pressure 101/53 mmHg, heart rate 143 beats/minute, respiratory rate 34 breaths/minute, oxygen saturation 94% on room air, and his temperature was 102.4 F. Chest radiograph showed near-complete opacification of the right hemithorax. An emergency chest tube was placed by the ED team due to concern for impending tension pneumothorax. Thoracentesis yielded 2 liters of serosanguinous fluid, later found to be neutrophil predominant exudate.

**Figure 1:**
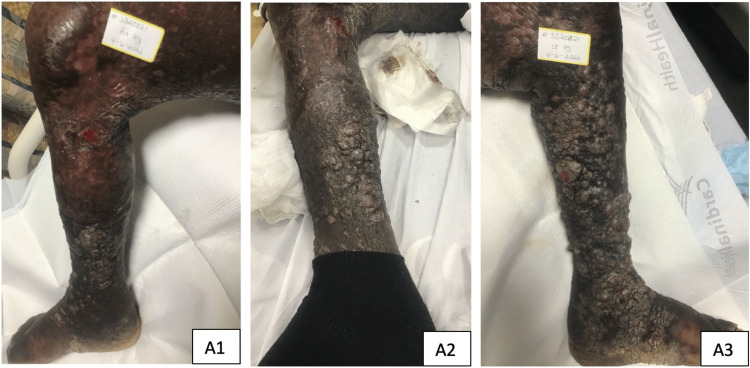
Multiple nodules of varying sizes, swelling, and crusting along distal lower extremities resembling possible elephantiasis demonstrated in A1, A2, A3.

Upon further inspection and examination of the lower extremities, the patient was found to have multiple hyperpigmented, erythematous, and friable nodules showing a confluence of violaceous patches and plaques of varying sizes, swelling, and crusting with ulcerated and eroded areas along distal lower extremities resembling possible elephantiasis presentation (Figure 1). There was no documentation of KS biopsy upon chart review. Dermatology was consulted and they performed a biopsy of one of the lesions which demonstrated papillomatosis with hyperkeratosis and dilated lymphatic channels, confirming KS with overlying ENV. Ammonium lactate topical lotion was applied daily which helped reduce nodular lesions of the lower extremities. Xeroform gauze, Telfa dressing, and bandages were applied to ulcerated/eroded lesions on the patient’s leg.

**Figure 2:**
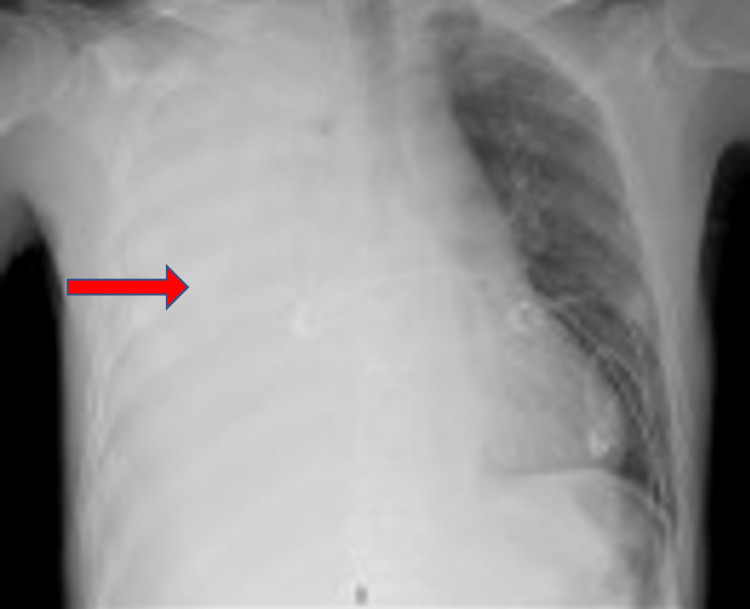
Portable (AP) chest X-ray shows right-sided pleural effusion with near-complete opacification of the right hemithorax shown by the red arrow.

**Figure 3:**
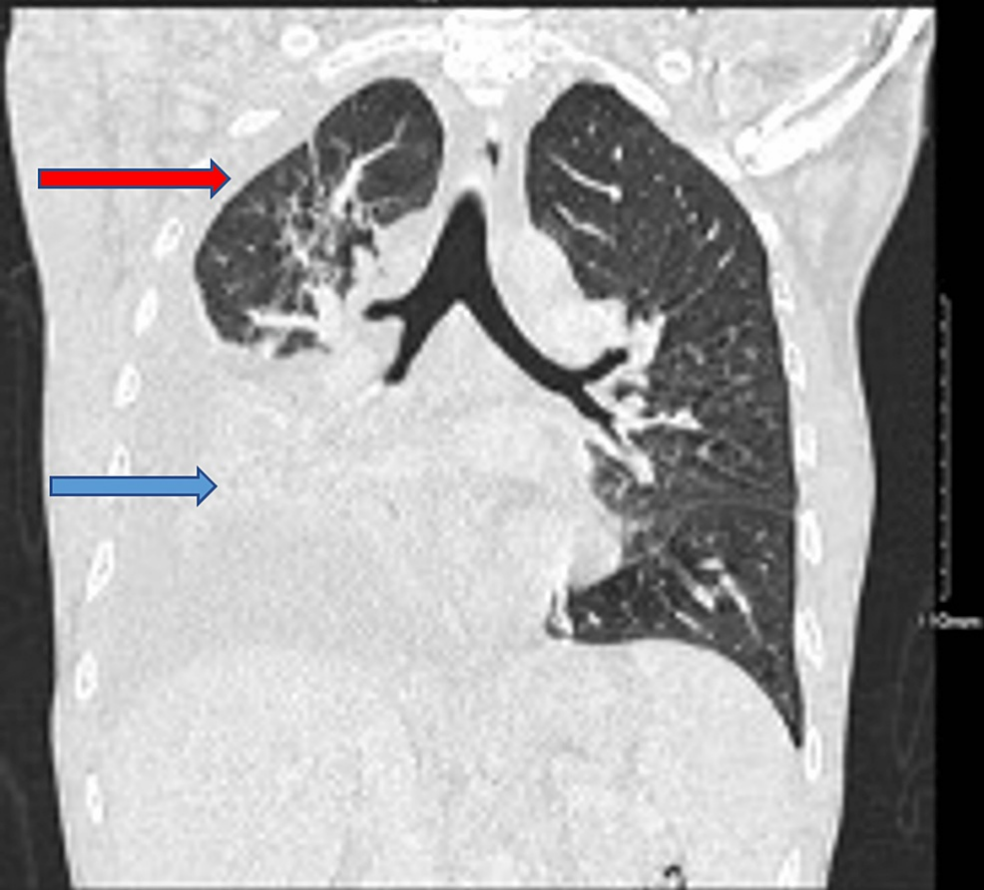
Chest CT shows right-sided consolidation depicted by the blue arrow and thickened pleura shown by the red arrow.

On day three of the hospital course, he developed worsening shortness of breath, requiring 15L of oxygen via a non-rebreather mask. A repeat chest radiograph at that time showed recurrent, right-sided pleural effusion with near-complete opacification of the right hemithorax (Figure 2). A chest-CT was performed which showed right-sided consolidation and thickened pleura (Figure 3).

Pleural fluid was successfully removed, and the patient underwent a video-assisted thoracoscopic surgery (VATS) with pleural biopsy. The pleural biopsy was negative for malignancy, suggesting that the pleural effusion was likely due to an infectious etiology which was treated empirically with ceftriaxone and doxycycline with clinical improvement. Subsequently, the patient developed a pneumothorax requiring a chest tube to suction. Unfortunately, the patient developed repeated pneumothoraces off suction, requiring the thoracic surgery team to perform pleurodesis.

Lastly, we observed that the patient was persistently hyponatremic throughout the admission, although hemodynamically stable and asymptomatic (Table 1). A complete workup revealed that it was likely secondary to trimethoprim-sulfamethoxazole use. The patient was switched to atovaquone and the sodium level was corrected. The patient continued to be treated with bictegravir/emtricitabine/tenofovir alafenamide (Biktarvy) and atovaquone throughout the hospital course.

**Table 1:** Laboratory values obtained throughout the hospital course.

	Day 1	Day 2	Day 3	Day 4	Day 5	Day 6	Day 7	Day 8	Day 9	Day 10	Day 11	Day 12	Day 13	Day 14	References
White blood cell	17.22	12.08	9.18	7.58	6.34	7.25	7.47	6.68	6.91	7.63	6.75	7.53	7.05	7.95	3.50 - 10.80 K/uL
Hemoglobin	10.5	6.9	7.7	8.6	8.5	7.4	6.9	7.4	7.3	7.2	7	8.5	8.1	8.3	14 - 18 g/dL
Platelet count	256	174	199	185	164	174	313	275	319	335	350	332	338	314	130 - 400 K/uL
Serum sodium	130	128	130	129	128	129	133	135	137	138	136	137	137	136	136 - 145 mmol/L
Serum potassium	4.4	4	4.2	4	4.2	4	4	3.9	4	4.3	4.5	5	4.9	4.6	3.5 - 5.1 mmol/L
Serum chloride	99	99	101	99	97	98	98	103	103	103	104	104	105	101	98 - 107 mmol/L
Serum bicarbonate	21	19	19	19	22	23	25	21	25	25	26	24	26	25	21 - 31 mmol/L
Serum blood urea nitrogen	11	14	13	12	8	9	17	17	15	13	15	21	22	14	7 - 25 mg/dL
Serum creatinine	1.07	1.23	1.05	0.95	0.79	0.7	0.85	0.77	0.75	0.65	0.76	0.82	0.72	0.88	0.7 - 1.3 mg/dL
Serum albumin	2.6	2.3	2	2.4	2.3	2.2	2.1	2	2	2.1	2.1	2.5	2.5	2.5	3.4 to 5.4 g/dL
Aspartate aminotransferase	11	13	8	9	11	9	11	11	10	14	15	13	14	15	< 37 U/L
Alanine aminotransferase	<5	<5	<5	5	<5	<5	<5	<5	<5	<5	<5	<5	<5	<5	< 55 U/L
Alkaline phosphatase	83	81	75	81	73	80	85	91	99	92	107	96	108	107	< 130 U/L
Venous Blood Gas pH	7.34														7.32 - 7.43
Urine analysis pH	5.5														5.0 - 7.0
COVID-19 PCR	Not detected														Not Detected
Influenza Type A	Not detected														Not Detected
Influenza Type B	Not detected														Not Detected

## Discussion

In this case report, we described a patient with known HIV/AIDS and KS who had acute on chronic respiratory distress due to recurring pleural effusions likely secondary to infectious etiology and negative for malignancy. He also presented with active serosanguinous drainage from multiple hyperpigmented, erythematous, and friable nodules showing a confluence of violaceous patches and plaques of varying sizes, swelling, and crusting with ulcerated and eroded areas on both lower extremities leading to the suspicion of overlying ENV. Although rare, the clinical presentation alongside the biopsy of a lesion demonstrating papillomatosis with hyperkeratosis and dilated lymphatic channels confirmed the diagnosis of ENV in this patient with AIDS-associated KS. Typically, cutaneous lesions are considered progressive manifestations of KS; however, when coupled with lymphedema and severe disfigurement, non-filarial elephantiasis should be considered due to chronic lymphatic obstruction.

Lymphoedema is due to the lymphatic load exceeding the transport capacity of the lymphatic system which causes fluid to accumulate in the interstitium. Due to impaired lymphatic drainage, the gravity-dependent area will enlarge, and hypertrophy can worsen with infection and inflammation [5]. The accumulation of protein-rich fluid in the interstitium is thought to cause a chronic inflammatory state, resulting in the transformation of soft tissue into hard fibrotic tissue as fibroblast, adipocytes, and keratinocytes accumulate. Histologic changes include hyperkeratosis, papillomatosis, verrucous hyperplasia, and adipocyte proliferation [6]. Other histologic changes may include loss of dermal papillae, fibrosis of the dermis and subcutaneous tissues, and widened lymphatic vessels [5]. As defined by the International Society of Lymphology, lymphostatic elephantiasis represents the most severe stage of progression of chronic lymphedema [6].

In the case of KS herpesvirus, the exact mechanism by which lymphedema arises is unclear. KS herpesvirus has genetic homologs of cyclin D, G-protein-coupled protein, interleukin-6, and macrophage inflammatory protein-1 and -2 which can mimic and disrupt host cytokine signals [7]. KS is also associated with upregulated cyclooxygenase-2 expression in infected cells, which contributes to inflammation and lesion pathogenesis through upregulating infected cell survival and inducing cytokines and angiogenic factors [8]. Consequently, lymphedema in the setting of KS may be a result of a vigorous inflammatory response [9].

Another possibility is that the cutaneous lesions in KS can cause lymphatic obstruction through external compression. Other research suggests that direct communications between cutaneous lesions and lymphatics alongside intermixing of blood and lymph may contribute to lymphedema in the setting of KS [10]. Furthermore, some studies have suggested that KS herpesvirus may reprogram the vascular endothelium to express main lymphatic lineage-specific genes including lymphatic vessel endothelial receptor 1 (LYVE1), podoplanin, and vascular endothelial growth factor receptor 3 (VEGFR3) [1,11].

The treatment of ENV is challenging due to the lack of literature surrounding the management of elephantiasis. Ultimately, the goal is to treat the underlying cause of the lymphedema which in this case is KS. Supportive treatment of ENV includes elevation, massage, multilayer inelastic lymphedema bandaging, and compression stockings. More intensive therapy includes decongestive lymphatic therapy. Sequential pneumatic compression of extremity lymphedema is another possible treatment option. Still, it must be used early in the disease course as studies have shown that patients with fibrotic lymphedema respond poorly. Pharmacologic options include oral or topical retinoids as an adjunct to physiotherapy, diuretics, or coumarin (benzopyrone) which may produce limited success in reducing tissue volume, topical keratolytic (eg, salicylic acid) as well as deodorant powders to manage odor. Skin integrity must also be preserved to reduce the chance of bacterial or fungal infection which can be achieved through the use of emollients to prevent desiccation and breakdown of skin. If the skin does break down, topical antimicrobial agents may be considered to prevent life-threatening sepsis, especially in the context of an immunosuppressed patient such as the patient being presented. Lastly, surgical treatment should be considered after a medical intervention has already been attempted. Surgical debridement may provide comfort by reducing the verrucous lesions, and limb amputation can be considered for ENV that does not respond to treatment [6,10]. Interestingly, the CO2 laser has successfully treated ENV recalcitrant to topical therapy in a handful of case reports [12].

KS lesions correlated with lymphedema are considered a poor prognostic risk factor in the staging of KS in AIDS [9]. Improving lymphatic drainage is essential in avoiding the significant morbidity and irreversible fibrosis of subcutaneous tissue that is associated with lymphedema [10]. 

In this case report, we described a patient with known HIV/AIDS and KS who had acute on chronic respiratory distress due to recurring pleural effusions. He also presented with active serosanguinous drainage from large nodular lesions on both lower extremities leading to the suspicion of overlying ENV. Although rare, workup led to the diagnosis of ENV in this patient with AIDS-associated KS. Typically, cutaneous lesions are considered progressive manifestations of KS; however, when coupled with lymphedema and severe disfigurement, non-filarial elephantiasis should be considered due to chronic lymphatic obstruction. This case report demonstrates that although rare, ENV must be considered as a possible complication in a patient with AIDS-associated KS.

## Conclusions

Non-filarial elephantiasis, ENV, is very rare but can be found in a patient with known AIDS-associated KS. It occurs due to chronic lymphatic obstruction causing lymphedema and progressive cutaneous hypertrophy. This case report emphasizes the importance of a thorough physical exam, having a broad differential diagnosis, and workup for ENV in AIDS-associated KS patients who present with severe disfigurement.

In patients who present with AIDS-associated KS, ENV should be assessed if lymphedema is present. Symptoms and complications of the low-grade vascular tumor can be severe in patients with HIV/AIDS with severe disfigurement, and mucosal and visceral involvement. ENV is a very rare presentation in a patient with KS, occurring due to chronic lymphedema.
